# Feasibility and advantage of adding ^131^I-MIBG to ^90^Y-DOTATOC for treatment of patients with advanced stage neuroendocrine tumors

**DOI:** 10.1186/s13550-014-0038-2

**Published:** 2014-09-10

**Authors:** David L Bushnell, Mark T Madsen, Thomas O’cdorisio, Yusuf Menda, Saima Muzahir, Randi Ryan, M Sue O’dorisio

**Affiliations:** Department of Radiology, Division of Nuclear Medicine, University of Iowa Hospitals and Clinics, Iowa City, IA 52242 USA; Diagnostic Imaging Service, Iowa City Veterans Administration Medical Center, Hwy 6, Iowa City, IA 52242 USA; Department of Internal Medicine, Division of Endocrinology, University of Iowa Hospitals and Clinics, Iowa City, IA 52242 USA; University of Iowa College of Medicine, Iowa City, IA 52242 USA; Department of Pediatrics, Division of Oncology, University of Iowa Hospitals and Clinics, Iowa City, IA 52242 USA

**Keywords:** ^90^Y-DOTATOC, ^131^I-MIBG, Radionuclide theranostics, Neuroendocrine tumors

## Abstract

**Background:**

Peptide receptor radionuclide therapy (PRRT) is an effective form of treatment for patients with metastatic neuroendocrine tumors (NETs). However, delivering sufficient radiation dose to the tumor to result in a high percentage of long-term tumor remissions remains challenging because of the limits imposed on administered activity levels by radiation damage to normal tissues. The goal of this study was to evaluate the dosimetric advantages of adding ^131^I meta-iodobenzylguanidine (^131^I-MIBG) to ^90^Y DOTA Phe1-Tyr3-octreotide (^90^Y-DOTATOC) in patients with advanced stage midgut NETs.

**Methods:**

Ten patients were imaged simultaneously with ^131^I-MIBG and ^111^In-pentetreotide (as a surrogate for ^90^Y-DOTATOC) on days 1, 2, and 3 post-administration. Blood samples were obtained at the same time points. Using dosimetry measures from this data and our previously published methodology for calculating optimal combined administered activity levels for therapy, we determined the amount of ^131^I-MIBG that could be added to ^90^Y-DOTATOC without exceeding normal organ dose limits (marrow and kidneys) along with the expected increase in associated tumor dose, if any.

**Results:**

We found that a median value of 34.6 GBq of ^131^I-MIBG could be safely added to ^90^Y-DOTATOC (delivered over multiple cycles) by reducing the maximum total deliverable ^90^Y-DOTATOC by a median value of 24.5%. Taking this treatment approach, we found that there would be a median increase in deliverable tumor dose of 4,046 cGy in six of the ten subjects. Of note, there were a small number of metastases that were positive for only one or the other of these radiopharmaceuticals within the same subject.

**Conclusions:**

We conclude that approximately half of the patients with midgut NETs that are eligible for PRRT could reasonably be expected to benefit from the addition of ^131^I-MIBG to ^90^Y-DOTATOC.

## Background

Peptide receptor radionuclide therapy (PRRT) is well established as an effective form of treatment for patients with metastatic neuroendocrine tumors (NETs) delivering modest objective response rates but notable symptomatic and probable survival benefits [1–3]. PRRT with either ^90^Y DOTA Phe1-Tyr3-octreotide (^90^Y-DOTATOC) or the alternative ^177^Lu DOTA Phe1-Tyr3-octreotate (^177^Lu-DOTATATE) is recommended by the European and North American neuroendocrine tumor societies for the treatment of patients with non-operable refractory disease [4–6] ^90^Y-DOTATOC has shown significant efficacy as a therapeutic agent in patients with metastatic neuroendocrine tumors [7–10]. However, delivering sufficient radiation dose to the tumors to result in a high percentage of long-term remissions remains challenging because of the limits imposed on administered activity levels by radiation damage to normal tissues. For ^90^Y-DOTATOC, the radiation dose to kidneys limits the level of activity that can be administered to a given patient.

^131^I meta-iodobenzylguanidine (^131^I-MIBG) is well known as an effective form of therapy for advanced stage pheochromocytomes or neuroblastomas [11,12]. In addition, there is good evidence that ^131^I-MIBG can be an effective treatment for certain patients with gastroenteropancreatic NETs [13,14]. Significant tumor uptake of MIBG is reported in over 50% of patients with NETs of midgut origin [15–17]. In contrast to ^90^Y-DOTATOC, toxicity to bone marrow limits the level of administered activity that can be delivered with ^131^I-MIBG. In addition, the tumor targeting mechanism is distinctly different for MIBG. We have previously demonstrated both conceptually and mathematically that this difference in the toxicity and biodistribution profiles for these two radioactive drugs allows us to combine large fractions of the individually deliverable maximum tolerated administered activity of each radioactive drug into a single treatment regimen yielding potentially higher tumor radiation doses without exceeding normal organ dose limits [18,19].

There are three potential advantages to adding ^131^I-MIBG to PRRT with ^90^Y-DOTATOC for the treatment of neuroendocrine tumors as opposed to using PRRT alone. First of all, as noted above, our preliminary work indicates that it should be possible to notably increase the delivered tumor radiation dose beyond that achievable with ^90^Y-DOTATOC alone in some patients with neuroendocrine tumors that also concentrate MIBG [18]. Secondly, because of the different tumor targeting mechanisms for MIBG and octreotide, it may be possible to achieve therapeutic radiation delivery to a greater number of tumor cells or tumor sites than with only ^90^Y-DOTATOC [20,21]. And thirdly, studies have demonstrated that there is an advantage to using a combination of radioactive drugs that have different beta particle energies to treat metastatic lesions when they vary significantly in size [22].

The overall goal of this study was to investigate the feasibility and advantages of combining ^131^I-MIBG with ^90^Y-DOTATOC to treat NETs compared to treatment with ^90^Y-DOTATOC alone through the use of pre-therapy biodistribution and dosimetry results. The importance of this type of approach using the theranostics concept to manage patients with NETs has been recently emphasized [23].

The specific goals were (1) to determine in what fraction of patients with midgut NETs a substantial amount of ^131^I-MIBG could be safely added to ^90^Y-DOTATOC without exceeding normal organ dose limits, (2) to determine whether tumor radiation dose levels could be increased by more than 30% through addition of ^131^I-MIBG to ^90^Y-DOTATOC, and (3) to determine if additional tumor sites would be targeted through the addition of ^131^I-MIBG.

## Methods

This was a prospective study of patients with metastatic or otherwise non-operable neuroendocrine tumors of midgut origin. All patients were required to have a serum creatinine level of less than 2.0 mg/dl and were excluded if they had undergone prior PRRT.

All subjects underwent imaging (and blood sampling) with tracer amounts of ^131^I-MIBG and ^111^In-pentetreotide for purposes of determining individualized radiation dose levels per unit of administered activity to the kidneys, marrow, and select tumor sites. Patients were not allowed to use their short-acting Sandostatin (Sandoz GmbH, Schaftenau, Austria) beginning 12 h prior to day 1 of the study until completion of imaging and were required to be at least 21 days out from their last Sandostatin LAR injection (Sandoz GmbH). Subjects did not receive cationic amino acid infusions. Rather, we built into our model a 20% reduction in renal radiation dose from ^90^Y-DOTATOC reflecting what is known to occur with the use of a cationic amino acid infusion during an actual ^90^Y-DOTATOC treatment [24].

Each subject underwent multiple imaging sessions over a 3-day period following radiopharmaceutical administration as outlined in Table 1. Each subject received 0.5 mCi (first two subjects enrolled) or 1.0 mCi of ^131^I-MIBG plus 5 to 6 mCi of ^111^In-pentetreotide intravenously within a few minutes of each other. All scintigraphic imaging studies were acquired as multi-isotope studies with a 20% window on the 364-keV photopeak of ^131^I and the 247- and 172-keV photopeaks of ^111^In. High energy collimation, appropriate for ^131^I, was used for all simultaneous imaging studies. Anterior and posterior planar whole-body images along with single photon emission computed tomography (SPECT) studies of the chest/abdomen/pelvis were obtained at (nominally) 4, 24, and 48 h after injection. Beyond 48 h, clearance data for blood, kidneys, and selected tumors was based on extrapolation of the curve from the 24- and 48-h time points. Standard sources of ^131^I and ^111^In with an activity of 1 to 2 mBq were placed within the field of view at each image acquisition session. Contrast enhanced computed tomography (CT) images of the chest, abdomen, and pelvis were obtained if the subject had not had a recent diagnostic CT exam (within 6 months).

**Table 1:** **Imaging/biodistribution data collection schedule**

	**Day 0**	**Day 1**	**Day 2**
Radiopharmaceutical injection (^131^I-MIBG and ^111^In-pentetreotide)	X		
Blood samples	X (1 and 4 h)	X	X
Whole-body conjugate views	X	X	X
SPECT	X	X	X

Corrections for down-scatter were performed for the image data as well as the blood samples assayed on the well counter. For the well counter measurements, the down-scatter fraction of counts from the 131I 364-keV gamma rays into the ^111^In energy windows was determined to be 0.25. An additional up-scatter component correction (0.67) had to be made because of the strong coincidence sum peak associated with the In-111 172- and 254-keV cascade. For the image data, the down-scatter fraction was approximately 0.15. Because of the high ratio of administered activity of ^111^In-pentetreotide to ^131^I-MIBG and the much higher detection efficiency for the ^111^In gamma rays, down-scatter corrections were essentially negligible.

The patient-specific kinetic and biodistribution results from the concurrent tracer studies with ^131^I-MIBG and ^111^In-pentetreotide were then used to calculate values for radiation dose per total administered activity (mGy/MBq) for marrow, kidneys, and selected tumors for both ^131^I-MIBG and ^90^Y-DOTATOC. Dosimetry calculations were based on the conjugate view methodology for kidneys and tumor sites applying patient-specific renal mass as measured by CT for each individual. CT was also used to determine tumor volume/mass. A representative soft tissue metastasis with well-defined borders of at least 2 cm in diameter on CT was selected from each subject for dosimetry calculations when there was visible tumor uptake with *both*^131^I-MIBG and ^111^In-pentetreotide. Bone marrow dose calculations were based on clearance and activity measures from the blood samples. We assumed a 1:1 ratio of activity in the blood relative to the bone marrow for each radiopharmaceutical. The tumor, blood, and kidney doses were estimated using first principle methods originally described by Quimby and incorporated into the MIRD/OLINDA formulations [25]. The total integrated activity was multiplied by the average emitted energy modified by the appropriate absorption factors divided by the tissue mass. For dose estimates with ^90^Y and the beta component of ^131^I, the absorbed fraction was assumed to be 1 for activity located within the tissue under consideration and 0 from all external sources.

The patient-specific dosimetry values for marrow and kidneys for each subject were then used with our previously described methods to calculate the maximum safe administrable activity for ^90^Y-DOTATOC when given alone and then for each of the two radiopharmaceuticals when given in combination using previously described methods [18]. To make these calculations requires setting upper limits for total radiation dose to an individual's bone marrow and kidneys from such a treatment. In this study, we used a limit of 2,300 cGy for the kidneys and 300 cGy for bone marrow. Although 300 cGy for a marrow upper limit would be high for a single non-myeloablative treatment, in practice, this dose would be spread over two or three individual treatment cycles (every 8 to 10 weeks to allow for marrow recovery) leading to a marrow dose per cycle of a very reasonable 100 to 150 cGy. Finally, adding the tumor dosimetry results to the patient's renal and marrow results in the model allowed us to calculate the expected increase, if any, in tumor dose from a combined therapy.

## Results

There were a total of ten subjects in this study. The median patient age was 58 years (range 37 to 76 years). There were six men and four women. All subjects had confirmed midgut NETs. At least one soft tissue metastatic lesion was present in all subjects and identifiable on corresponding CT upon entry into the study. Nine of the ten subjects had metastatic disease in the liver, while one individual had metastatic disease present only in two large abdominal nodal masses. One patient with liver metastases also had a metastatic lung lesion, and another patient with liver metastases had metastatic lesions in both breasts.

**Figure 1:**
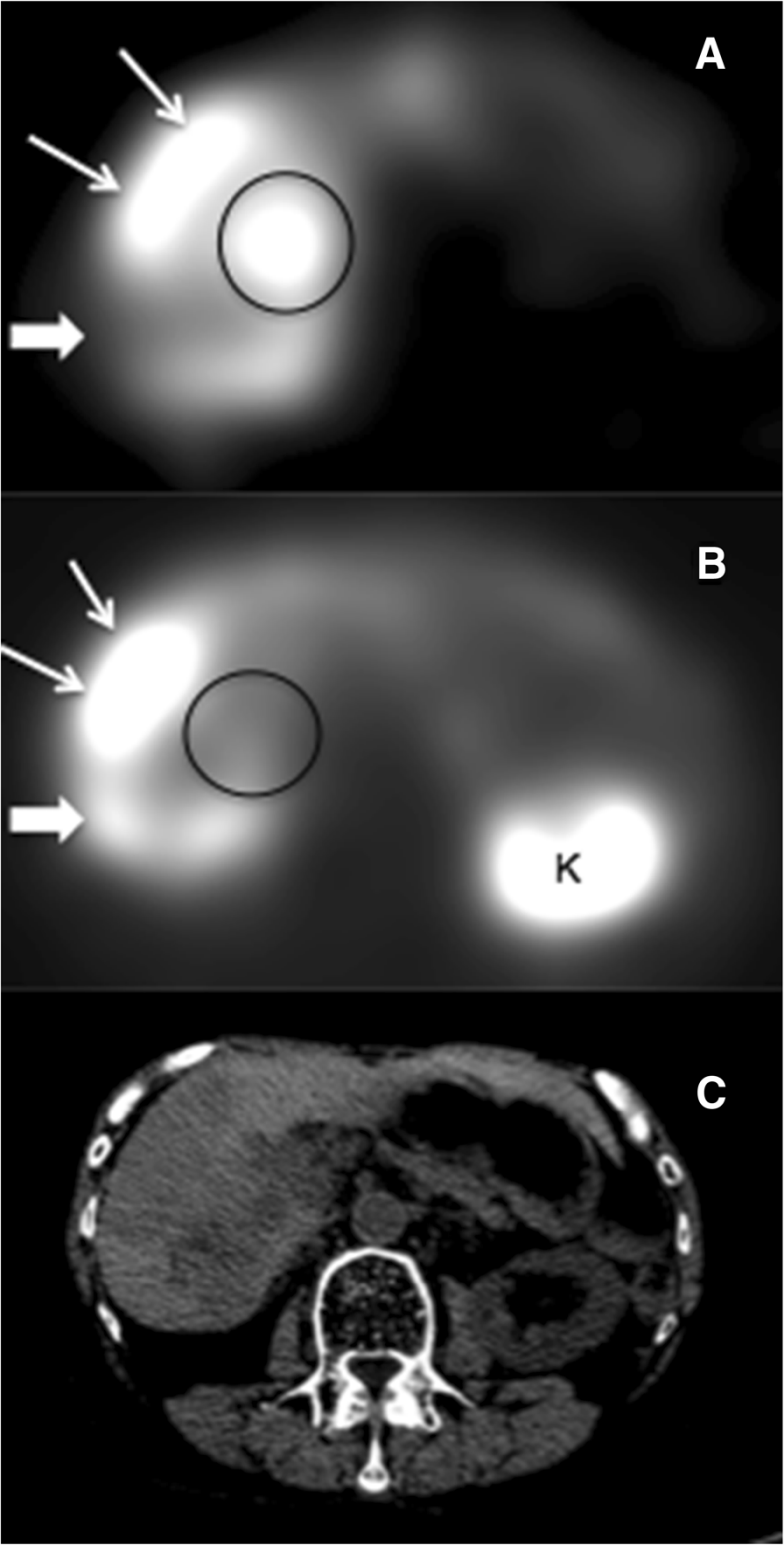
**Example of discordant** ^**131**^ **I-MIBG and** ^**111**^ **In-pentetreotide tumor uptake.** SPECT ^131^I-MIBG **(A)** and ^111^In-pentetreotide **(B)** images and corresponding CT **(C)** at the same level from subject #6 showing a large metastatic lesion that is both MIBG and pentetreotide positive (thin arrows), along with a metastasis which is MIBG positive and pentetreotide negative (black circle) and another that is pentetreotide positive and MIBG negative (thick arrow).

Planar and SPECT ^111^In-pentetreotide images were positive for at least one metastatic site in all ten subjects. ^131^I-MIBG planar and SPECT imaging were both positive for metastatic disease in six of the ten subjects. Tumor dosimetry was therefore performed in these six subjects, specifically on a liver metastasis in four subjects and on an abdominal nodal mass in one subject and a large breast metastasis in one subject. ^131^I-MIBG and ^111^In-pentetreotide tumor uptake pattern concordance (or discordance) for each subject is given in the first column of Table 2. Table 3 provides a summary of individual lesion MIBG and pentetreotide uptake status for these six subjects. As seen in this table, the majority of the soft tissue metastases were visually positive with both agents. However, there were a notable number of lesions that were positive with MIBG only and showed a pattern of complete concordance for metastatic disease with the corresponding ^111^In-pentetreotide images in two of these six subjects. In the other four cases, the pattern of tumor uptake was modestly discordant not only showing metastases (predominately hepatic) that were MIBG positive and octreotide positive but also showing 1 to 2 tumor sites in each case that were ^131^I-MIBG positive but ^111^In-pentetreotide negative and vice versa. Figure 1 depicts one of the study patients with multiple hepatic metastases, the majority of which concentrated both radiopharmaceuticals. However, this individual also had two hepatic lesions which only demonstrated uptake with MIBG (one of which is depicted in the image). Excluding the 4 subjects where MIBG imaging was entirely negative, there were a total of 7 MIBG-positive/octreotide-negative lesions, 8 MIBG-negative/octreotide-positive lesions, and 22 MIBG-positive/octreotide-positive metastatic lesions.

**Table 2:** **Tumor dose and administered activity results for the addition of** ^**131**^ **I-MIBG to** ^**90**^ **Y-DOTATOC**

**Subject**	**MIBG vs. pentetreotide image pattern for metastases**	**Maximum activity (GBq)** ^**90**^ **Y-DOTA alone (over multiple cycles)**	**Optimum percentage of max** ^**90**^ **Y-DOTA activity to be given when adding MIBG**	**Activity (GBq) of** ^**131**^ **I-MIBG that can be added without exceeding limits (over multiple cycles)**	**Tumor dose (cGy):** ^**90**^ **Y-DOTA given alone**	**Tumor dose (cGy):** ^**90**^ **Y-DOTA +** ^**131**^ **I-MIBG**
1	Concordant	13.5	72	32.2	1,623	7,618
2	All tumors MIBG negative					
3	All tumors MIBG negative					
4	Discordant	11.1	88	29.8	3,317	11,119
5	All tumors MIBG negative					
6	Discordant	14.4	16	42.8	10,330	45,737
7	Discordant	19.8	100	0.0	14,068	14,068
8	Discordant	4.9	75	33.3	1,006	2,499
9	Concordant	7.2	76	45.0	729	2,829
10	All tumors MIBG negative					
Calculated using average dosimetry results from the literature^a^	N/A	10.7	85	36.4	4,685	14,555

Total renal limit, 23 Gy; total marrow limit, 3 Gy. N/A, not applicable. ^a^Results in this row were calculated using average dose values for the marrow, kidneys, and NETs from the literature.

**Table 3:** **MIBG and pentetreotide individual lesion uptake pattern**

	**MIBG +**	**MIBG −**
Pentetreotide **+**	22	8
Pentetreotide **−**	7	2^a^

^a^Seen on CT only.

For the six MIBG-positive subjects, the mean value for maximum administrable activity of ^90^Y-DOTATOC was found to be 11.8 GBq (standard deviation (S.D.) = 4.9 GBq). Again, in practice, this total activity would be delivered over several individual treatment cycles. We calculated that it would be ‘safe’ (not exceed normal organ limits) for these six subjects to concurrently receive a mean value of 30.0 GBq (S.D. = 14.8 GBq) of ^131^I-MIBG by reducing the total administered ^90^Y-DOTATOC activity by a mean value of 26% (S.D. = 27%). The increase in tumor dose to a representative tumor site achievable by adding ^131^I-MIBG to ^90^Y-DOTATOC in these six subjects ranged from 0 to 35,407 cGy with a median increase of 4,046 cGy and mean increase of 8,799 cGy (S.D. = 12,196 cGy). This additional tumor dose represents an increase of 169% over the mean tumor dose calculated for maximum ^90^Y-DOTATOC when given alone (5,180 cGy). The data for each individual subject is presented in Table 2. We also performed the calculations using reported dosimetry results from the literature for the kidney, marrow, and tumor for ^131^I-MIBG and ^90^Y-DOTATOC [26–29]. These results are also given in the last row of Table 2. Table 4 shows the effect on the calculated results of changing normal organ dose limits for a given subject (#8). As can be seen from this example, reducing the limit for marrow exposure to 200 cGy from 300 cGy has a notable effect on the magnitude of the benefit of adding ^131^MIBG to PRRT. Specifically, the calculated level of ^131^I-MIBG activity that can be ‘safely’ added to ^90^Y-DOTATOC drops from 33.3 to 17.6 GBq for this subject. Accordingly, the tumor dose benefit drops from 1,493 to 788 cGy. In this same case, leaving the marrow limit at 300 cGy but increasing the renal dose limit to 2,700 cGy (as some investigators have suggested is reasonable) has only a small effect on the calculated results as depicted in the last row of this table.

**Table 4:** **Results using different normal organ dose limits for subject #8**

	**Organ dose limits**	**Maximum activity (GBq)** ^**90**^ **Y-DOTA alone (given over multiple cycles)**	**Optimum percentage of maximum** ^**90**^ **Y activity to be given when adding MIBG**	**Acivity (GBq) of** ^**131**^ **I-MIBG that can be added without exceeding limits**	**Tumor dose (cGy):** ^**90**^ **Y-DOTA given alone**	**Tumor dose (cGy):** ^**90**^ **Y +** ^**131**^ **I**
Kidney	2,300	4.9	75	33.3	1,006	2,499
Marrow	300					
Kidney	2,300	4.9	87	17.6	1,006	1,794
Marrow	200					
Kidney	2,700	5.8	80	30.8	1,180	2,565
Marrow	300					

## Discussion

In essence, our methodology and concept boils down to this: By measuring these following variables for a given patient,

- m_MIBG_ = ^131^I-MIBG red marrow dose per megabecquerel
- m_DOTA_ = ^90^Y-DOTATOC red marrow dose per megabecquerel
- k_MIBG_ = ^131^I-MIBG kidney dose per megabecquerel
- k_DOTA_ = ^90^Y-DOTATOC kidney dose per megabecquerel,

we are able to calculate the optimal amount of ^131^I-MIBG to add to the optimal amount of ^90^Y-DOTATOC that will not exceed the pre-defined kidney and marrow dose limits. Furthermore, by measuring these additional patient-specific variables,

- t_MIBG_ = ^131^I-MIBG tumor dose per megabecquerel
- t_DOTA_ = ^90^Y-DOTATOC tumor dose per megabecquerel,

we are able to calculate the expected increase in tumor dose, if any, that would occur by adding the calculated amount of ^131^I-MIBG to ^90^Y-DOTATOC.

Results of this study strongly support a dosimetric benefit through the addition of ^131^I-MIBG to ^90^Y-DOTATOC. Although this requires the use of patient-specific dosimetry measurements, such an approach is consistent with an ever greater emphasis on individualized medicine. We have demonstrated that a mathematically definable level of ^131^I-MIBG can be added to ^90^Y-DOTATOC by reducing the maximum administered activity of ^90^Y-DOTATOC by a calculated amount yielding in most cases a substantial increase in delivered tumor dose while remaining within specified dose limits for bone marrow and kidneys. We found this to be the case in five of the ten total subjects that we studied and in five of the six subjects where MIBG showed visible tumor uptake. The one case out of these six where it was not true was somewhat unusual in that for this patient the kidneys turned out not to be the dosage limiting organ for ^90^Y-DOTATOC. In this patient, bone marrow radiation dose would have limited the maximum amount of administrable ^90^Y-DOTATOC. Specifically, we found that the group median increase in tumor dose achievable through the addition of ^131^I-MIBG was 4,046 cGy and the group mean increase was 8,799 cGy (albeit with a very large standard deviation). These results are large enough to suggest the potential for a meaningful amount of additional tumor cell kill.

Our results also corroborate findings from older reports suggesting some differences in tumor targeting patterns for MIBG versus octreopeptide within the same patient [20,21,30]. Specifically, we found a clear evidence of additional tumor site targeting with the addition of MIBG in four of the ten subjects in our study, although it should be kept in mind that the majority of metastatic lesions were positive with both agents in these four subjects. These results further support the value of adding ^131^I-MIBG to the treatment regimen for certain patients receiving PRRT. Our finding that MIBG is positive overall in 60% of midgut NET patients is also consistent with other reports [15–17].

The amount of additional ^131^I-MIBG activity that could be safely added to ^90^Y-DOTATOC along with the required fractional reduction in ^90^Y activity would change modestly if different normal organ (kidney and marrow) dose limits were applied as depicted in Table 3. For example, if the marrow limit was reduced, the additional amount of ^131^I-MIBG that could be added would also be reduced as shown in the table. Although 300 cGy to the marrow would be excessive for a single treatment, this number is a cumulative limit that would in practice be achieved through administration of the treatment drugs over several individual cycles (separated by 8 to 12 weeks to allow for marrow recovery). Going forward, there is also a possibility that normal organ limits would be better established using the biologically effective dose concept, and this may merit consideration for future investigations [31].

Because ^90^Y is almost entirely a pure beta emitter, it is quite difficult to obtain pre-therapy biokinetic imaging measurements for dosimetry calculations using ^90^Y-DOTATOC itself. Rather, a surrogate radiopharmaceutical with very similar pharmacokinetics must be used in practice. Other radiopharmaceuticals such as ^111^In-pentetreotide, ^111^In-DOTATOC, and ^86^Y-DOTATOC (PET) have been used as surrogate agents for this purpose [32,33]. ^111^In-pentetreotide, the commercially available agent we used in this project has been found to be a good, albeit not ideal, dosimetry surrogate for ^90^Y-DOTATOC [34]. Importantly, in a study using ^86^Y-DOTATOC PET as the gold standard for measuring renal residence times for ^90^Y-DOTATOC, Helisch and co-workers concluded that ^111^In-pentetreotide was a reasonable and acceptable surrogate for ^90^Y-DOTATOC when assessing individual patient pre-therapy renal dosimetry [35].

While the ^111^In-pentetreotide surrogate approach appears reasonably good for estimating renal and blood dosimetry for ^90^Y-DOTATOC, tumor dosimetry with this approach is likely somewhat less accurate. Almost certainly, ^111^In-pentetreotide tumor dosimetry will underestimate the actual delivered dose from ^90^Y-DOTATOC [33,36]. This is primarily because DOTATOC shows greater affinity for the overexpressed SST2 receptor than does pentetreotide [37]. While this would have an effect on our calculations of the tumor dose that can be delivered with ^90^Y-DOTATOC alone, it would not have an effect on our calculation of the absolute increase in tumor dose achievable through the addition of ^131^I-MIBG.

^177^Lu-DOTATATE is currently undergoing an international phase 3 trial and may become available for clinical use sooner than the PRRT alternative ^90^Y-DOTATOC. There is some evidence that ^177^Lu-DOTATATE may be even more effective than ^90^Y-DOTATOC although the only comparison of these two agents in the same study is from a recent retrospective report which found similar efficacy for the two radioactive drugs [38]. The concept of a dosimetric benefit through addition of ^131^I-MIBG to ^90^Y-DOTATOC could, at least qualitatively, be applicable to ^177^Lu-DOTATATE as well since the limiting organ for ^177^Lu-DOTATATE is also the kidney. However, there would be quantitative differences since renal and tumor dosimetry for ^90^Y-DOTATOC and ^177^Lu-DOTATATE, while similar, are not the same [39].

There is both pre-clinical and clinical data strongly supporting an enhanced effectiveness for the combination of ^177^Lu-DOTATOC plus ^90^Y-DOTATOC which is felt to be related to the greater spectrum of beta particle energies achievable when both agents are delivered together leading to better efficacy for a wider range of tumor sizes [22,40]. Since the beta energy spectrums for ^131^I and ^177^Lu are very similar, such a therapeutic advantage should also exist for the combination of ^131^I-MIBG and ^90^Y-DOTATOC (EBmax values for ^177^Lu, ^131^I, and ^90^Y are 0.5, 0.6 and 2.3 MeV, respectively).

There are limits to the accuracy of radiopharmaceutical dosimetry calculations in general and in our study specifically in that we utilized planar rather than SPECT/CT-based dosimetry for the kidneys and tumors. While the latter method is generally considered a more accurate technique for determining dosimetry for single photon emitters like ^131^I and ^111^In, the planar methodology is nevertheless time-honored and has been used by many investigators spanning a number of decades to determine both tumor and kidney dosimetry in studies with important clinical implications [23,41]. Furthermore, planar dosimetry measurements for kidneys in particular should be very reliable because kidneys are relatively large, usually have minimal overlying activity, and patient-specific renal mass was utilized in each calculation in our study. For practical purposes, patient participation was limited to a 3-day period. Because sampling was limited to 48 h post-injection, the clearance of the remaining activity in the tumor and normal tissues after 48 h was estimated from the calculated half time values from 24 to 48 h. Because of the 67-h half-life of ^111^In (and the closely associated 64 half-life of ^90^Y), this approach will be reasonably accurate since the physical half-life would limit any long biologic components. The results for ^131^I-MIBG could be affected to a greater degree, however, because the 8-day half-life would allow the expression of longer biologic components that were not sampled.

Nevertheless, it seems persuasive that our dosimetry results closely approximate those previously reported in the literature for ^131^I-MIBG and ^90^Y-DOTATOC [26,27,29]. Moreover, using an average of previously published dosimetry values for kidneys and marrow from other institutions, the calculated activity of ^131^I-MIBG that can be added to ^90^Y-DOTATOC without exceeding normal organ limits is quite similar to that which we calculated for the individual patients in our study (see the last row of Table 2).

## Conclusions

We conclude that approximately half of the patients with midgut NETs that are eligible for PRRT could benefit from the addition of ^131^I-MIBG to ^90^Y-DOTATOC. We believe the next step should be to test this approach in a phase 1 trial. While there might also be similar benefits for the addition of ^131^I-MIBG to ^177^Lu-DOTATATE, this possibility will require further investigation.

### Ethical standards

The protocol was approved by both the University of Iowa Hospitals and Clinics Investigational Review Board (IRB) and the Iowa City Veterans Administration Medical Center IRB, and all patients signed IRB-approved informed consent prior to entry into the study. This work has been performed in accordance with the ethical standards from the 1964 Declaration of Helsinki and its later amendments.
